# SIRT2 ablation inhibits glucose-stimulated insulin secretion through decreasing glycolytic flux

**DOI:** 10.7150/thno.55330

**Published:** 2021-03-04

**Authors:** Feiye Zhou, Linlin Zhang, Kecheng Zhu, Mengyao Bai, Yuqing Zhang, Qin Zhu, Shushu Wang, Chunxiang Sheng, Miaomiao Yuan, Yun Liu, Jieli Lu, Li Shao, Xiao Wang, Libin Zhou

**Affiliations:** 1 Department of Endocrine and Metabolic Diseases/ Shanghai institute of Endocrine and Metabolic Diseases, Ruijin Hospital, Shanghai Jiao Tong University School of Medicine, Shanghai 200025, China.; 2 Center for Reproductive Medicine, Shandong University, Jinan 250000, China.; 3 Department of VIP Clinic, Shanghai East Hospital, Tongji University School of Medicine, Shanghai 200123, China.

**Keywords:** SIRT2, islets, acetylation, glucokinase regulatory protein, glucose-stimulated insulin secretion, glycolysis.

## Abstract

**Rationale:** Sirtuins are NAD^+^-dependent protein deacylases known to have protective effects against age-related diseases such as diabetes, cancer, and neurodegenerative disease. SIRT2 is the only primarily cytoplasmic isoform and its overall role in glucose homeostasis remains uncertain.

**Methods:** SIRT2-knockout (KO) rats were constructed to evaluate the role of SIRT2 in glucose homeostasis. The effect of SIRT2 on β-cell function was detected by investigating the morphology, insulin secretion, and metabolomic state of islets. The deacetylation and stabilization of GKRP in β-cells by SIRT2 were determined by western blot, adenoviral infection, and immunoprecipitation.

**Results:** SIRT2-KO rats exhibited impaired glucose tolerance and glucose-stimulated insulin secretion (GSIS), without change in insulin sensitivity. SIRT2 deficiency or inhibition by AGK2 decreased GSIS in isolated rat islets, with lowered oxygen consumption rate. Adenovirus-mediated overexpression of SIRT2 enhanced insulin secretion from rat islets. Metabolomics analysis revealed a decrease in metabolites of glycolysis and tricarboxylic acid cycle in SIRT2-KO islets compared with control islets. Our study further demonstrated that glucokinase regulatory protein (GKRP), an endogenous inhibitor of glucokinase (GCK), was expressed in rat islets. SIRT2 overexpression deacetylated GKRP in INS-1 β-cells. SIRT2 knockout or inhibition elevated GKRP protein stability in islet β-cells, leading to an increase in the interaction of GKRP and GCK. On the contrary, SIRT2 inhibition promoted the protein degradation of ALDOA, a glycolytic enzyme.

**Conclusions:** SIRT2 ablation inhibits GSIS through blocking GKRP protein degradation and promoting ALDOA protein degradation, resulting in a decrease in glycolytic flux.

## Introduction

Protein lysine acetylation is a reversible post-translational modification that links acetyl-coenzyme A metabolism with cellular signaling 1. Recent advances in proteomics and mass spectrometry technology reveal that both histones and non-histone proteins are highly acetylated and constitute a major portion of the acetylome in mammalian cells 2. Indeed, acetylation is involved in key cellular processes, such as gene regulation, cell cycle, signal transduction, protein folding, autophagy, and metabolism 3. Notably, the majority of identified acetylated proteins are enzymes involved in energy metabolism, including glycolysis, tricarboxylic acid (TCA) cycle, electron transport chain, and fatty acid β oxidation 2, 4. This acetylation modification of metabolic proteins and enzymes occurs in response to altered nutrient status 5, 6, which regulates the balance between energy storage and expenditure 7. Pancreatic β-cells, extremely sensitive to nutrients alterations, secrete insulin at appropriate rates to maintain blood glucose levels within a relatively narrow range. Our recent study provides a comprehensive picture of protein acetylation in rat islets 8. But the direct effect of acetylation on islets function remains largely unknown.

Lysine acetyltransferases (KATs) and class I, II, III, and IV lysine deacetylases (KDACs) are responsible for reversible changes in protein acetylation status 9. Class III KDACs, termed sirtuins (SIRT1-SIRT7), require NAD^+^ as a substrate and keep highly conserved from bacteria to humans 10. Since the concentration of NAD^+^ is determined by the cellular nutritional state, sirtuins are considered to be metabolic and stress-sensor proteins that play important roles in energetic regulation. The deacetylase SIRT1, located in cell nuclei, is involved in carbohydrate and lipid metabolism by regulating key factors 11. SIRT1 activation ameliorates hyperglycemia by promoting insulin secretion and β-cell expansion 12, 13. SIRT3, SIRT4, and SIRT5 are mitochondrial sirtuins. SIRT3 targets key enzymes in fatty acid β oxidation pathway and regulates palmitate-stimulated insulin secretion 8, 14. SIRT4 regulates amino acid catabolism and maintains insulin secretion and glucose homeostasis during aging 15, 16. SIRT6 is also a predominantly nuclear isoform and has been implicated in a variety of metabolic processes 17. Pancreatic β-cell-specific SIRT6 knockout mice showed a significant decrease in glucose-stimulated insulin secretion (GSIS) 18.

Although SIRT2 is expressed in a wide range of tissues and organs, SIRT2 is still one of the least understood sirtuins. SIRT2 possesses efficient deacetylation activity and is the only primarily cytoplasmic isoform, which can also be found in the nucleus and mitochondria 19, 20. Previous studies for SIRT2 mainly focus on tumorigenesis and neurodegenerative diseases 21. Recently, accumulating evidence implicates the involvement of SIRT2 in various metabolic processes, including adipocyte differentiation, gluconeogenesis, and insulin sensitivity as well as inflammation 22. However, little is known about the role of SIRT2 in islet function.

In the present study, we generated SIRT2-knockout (KO) rats to determine its impact on glucose homeostasis. Impaired glucose tolerance and GSIS were observed in SIRT2-ablated rats. Metabolomics and oxygen consumption measurements revealed the involvement of glycolysis in SIRT2-regulated insulin secretion, in which SIRT2 inhibition decreased glucose-induced metabolic flux via stabilizing glucokinase regulatory protein (GKRP) and increasing its binding to glucokinase (GCK).

## Methods

### Experimental animals

Male Sprague-Dawley (SD) rats were purchased from Shanghai Laboratory Animal Company. All rats were group-housed in a barrier facility, receiving a regular chow diet ad libitum.

### CRISPR/Cas9-mediated SIRT2 knockout rats

For SIRT2 targeting, two sgRNAs were designed to target a region upstream of exon4 and downstream of exon7. sgRNAs were screened for on-target activity using the UCA kit and were successfully transcribed *in vitro*. Then Cas9/sgRNA plasmid and donor vector construction were completed. Cas9 mRNA, sgRNAs, and donor vector were mixed at proper concentrations and co-injected into the cytoplasm of fertilized eggs at one-cell stage. Surviving zygotes were transferred into the oviducts of pseudopregnant SD female rats (Beijing Biocytogen). Offspring rats were maintained on a Sprague-Dawley background and normal chow.

### Glucose and insulin tolerance tests

For glucose tolerance test (GTT), 8-week-old rats were injected with 2 g/kg body weight of glucose intraperitoneally after initial measurement of 16-h fasting blood glucose. Blood glucose concentrations were measured from the tail vein using a portable blood glucose meter (Lifescan, Johnson &Johnson) at 0, 15, 30, 60, and 120 min after the glucose injection. For insulin tolerance test (ITT), rats were injected with 0.75 IU/kg body weight of insulin intraperitoneally after initial measurement of 6-h fasting blood glucose. Blood glucose concentrations were measured at 0, 15, 30, 60, and 120 min after the insulin injection. Plasma was collected for insulin assay using ELISA kits (Mercodia). Total pancreatic insulin content was determined by radioimmunoassay, in which acidified ethanol extractions were performed on whole pancreases.

### Islet isolation and insulin secretion assay

Islets of Langerhans were isolated from 8- to 12-week-old SD male rats by collagenase digestion and density-gradient centrifugation as described previously 8. Before insulin secretion assay, isolated islets were cultured in RPMI 1640 medium (GIBCO) containing 5% fatal bovine serum (FBS, GIBCO) and 5 mM glucose for 4-6 h at 37 °C. Then, these islets were preincubated in Krebs-Ringer Buffer (KRB) containing 0.25% bovine serum albumin (BSA) and 3.3 mM glucose for 30 min. Ten islets per assay in triplicate were incubated with KRB buffer containing either various concentrations of glucose or the indicated reagents for a further 1 h at 37 °C. Supernatants containing insulin were collected for analysis. Insulin secretion was normalized to insulin content extracted with acidified ethanol. Insulin levels of all samples were measured by ELISA kit (Mercodia).

### Cell culture

INS-1 cells were cultured in RPMI 1640 medium containing 11.1 mM glucose, 10 mM HEPES, 10% FBS, 100 U/ml penicillin G sodium and 100 μg/ml streptomycin sulfate.

### Immunofluorescence staining

Pancreases were dissected, weighed, fixed in 4% formalin overnight at 4 °C, washed with phosphate buffered saline (PBS), and embedded in paraffin. The pancreatic sections were stained as previously described 23 using SIRT2 (Abcam, ab67299), insulin (Abcam, ab7842), and glucagon (Abcam, ab10988) antibodies. For α/β cell number statistics, 7-10 5 μm sections of pancreas (paraffin embedded, *n*=3 per genotype), separated by at least 200 μm, were co-stained for insulin and glucagon. The number of α/β cells per islet on the section level was determined by counting the glucagon^+^ /insulin^+^ cells manually.

INS-1 cells were fixed for 20 min in 4% formalin, permeabilized in 0.1% Triton×100 for 5 min, washed with PBS, and blocked in 5% BSA for 1 h. Cells were then incubated with anti-GKRP antibody (1: 100) overnight at 4 °C and stained with FITC-labeled IgG (1: 200, Jackson ImmunoResearch Laboratories). DAPI was added to stain cell nuclei. The cellular localization of GKRP was photographed and analyzed using a fluorescence microscope (LSM 880 with Airyscan, ZEISS).

### Adenovirus infection

For SIRT2/GKRP overexpression, vector adenovirus and adenovirus encoding wild-type rat SIRT2 or GKRP were transfected into islets or INS-1 cells for 48 h according to the manufacturer's instructions. The recombinant adenoviruses were generated by GeneChem (Shanghai, China).

### Western blotting and immunoprecipitation

Islets or cells were homogenized in RIPA buffer (20 mM Tris-HCl (pH 7.5), 150 mM NaCl, 1 mM EGTA, 1% NP-40, 1% sodium deoxycholate, 1 mM Na_3_VO_4_) containing protease inhibitor and phosphatase inhibitor (MCE). Protein lysates were separated by SDS-PAGE on 10% polyacrylamide gels and transferred to PVDF membranes (Millipore). Primary antibodies were detected with horseradish peroxidase (HRP)-conjugated secondary antibodies. Blotted membrane was imaged with a LAS-4000 Super CCD Remote Control Science Imaging System (Fuji). Anti-Flag was from Sigma-Aldrich. Anti-tubulin was from Proteintech. Anti-ATP5A, anti-UQCRC2, anti-MTCO1, anti-SDHB, and anti-rabbit/mouse IgG conjugated with HRP were purchased from Cell Signaling Technology. Anti-GCK, anti-GKRP, and anti-ALDOA were from Santa Cruz biotechnology. Anti-phosphoenolpyruvate carboxykinase (PEPCK) was from Abcam. Anti-HSP90 was from Millipore. Anti-acetyllysine was from PTM Biolab. Immunoprecipitation was performed by incubating protein lysates with the indicated antibodies for 2 h and then with protein A/G-agarose beads (Santa Cruz biotechnology) overnight at 4 °C. The immunoprecipitates were extensively washed with lysis buffer and eluted with SDS loading buffer by boiling for 10 min. Then, standard western blotting was followed.

### Real-time quantitative PCR

Total RNA was extracted from islets or cells by Trizol (Thermo Fisher). To quantify the transcript abundance of genes of interest, real-time quantitative PCR (RT-qPCR) was performed by using SYBR Green Premix Ex Taq (Takara) with an Applied Biosystems 7300 Real-Time PCR machine (Applied Biosystems).

### Islet metabolomics

The untargeted metabolomics profiling was performed on XploreMET platform (Metabo-Profile, Shanghai, China). For samples preparation, isolated islets from SIRT2-WT and SIRT2-KO male rats were incubated in KRB buffer with 16.7 mM glucose for 1 h. Then, these islets were washed twice with PBS and stored at -80 °C freezer before metabolomics analysis. A total of 1,000 islets from one genotype were pooled to create a uniform sample. Untargeted metabolomics samples were collected from six independent biological replicates.

### Oxygen consumption rate measurement

Islet oxygen consumption measurements were conducted on a Seahorse XFe24 analyser (Agilent Technologies). Islets were placed on a 24-well islet plate in XF Base Media (50 islets per well) with 3 mM glucose and equilibrated at 37 °C for 1 h in a CO_2_-free incubator. During the measurement of glucose-induced respiration, islets were treated with different concentrations of glucose and subsequently treated with carbonyl cyanide 4-(trifluoromethoxy) phenylhydrazone (FCCP, 5 µM) at the indicated time point. For glucose-induced ATP-coupled OCR, islets were treated with 16.7 mM glucose and 5 µM oligomycin in succession. Each analysis was replicated five times.

### Mitochondrial area and morphology analysis

INS-1 cells were seeded in a 96-well plate and subsequently treated with or without AGK2 for 4 h. Immunofluorescence dye Mito Tracker and hoechst were added into medium 15 min before the 96-well plate was moved into PerkinElmer Operetta CLS to obtain confocal images and the analysis of mitochondrial area and morphology.

### Statistics

Data were expressed as mean ± SEM. Comparisons were performed using ANOVA for multiple groups or the Student's t-test for two groups. Statistical significance was established at* P*< 0.05.

## Results

### Deletion of SIRT2 impairs glucose tolerance and insulin secretion in rats

**Figure 1:**
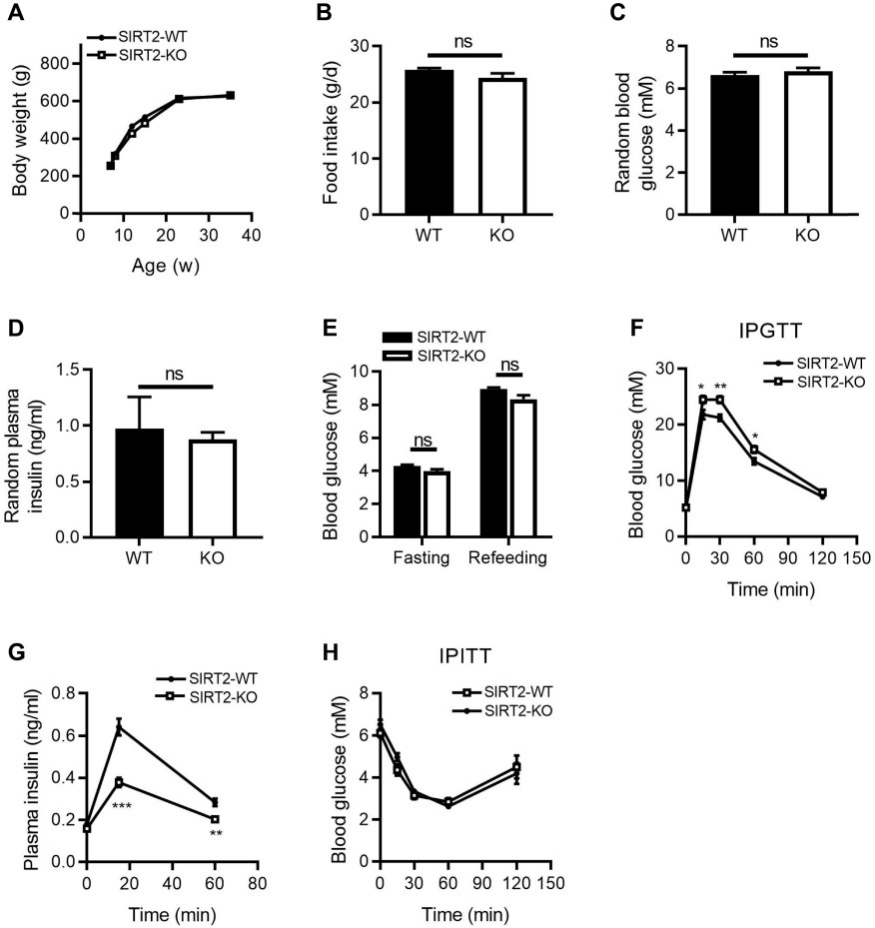
** Metabolic phenotypes of SIRT2-KO male rats. (A)** Body weight curves of SIRT2-WT and SIRT2-KO rats (*n*=8). **(B)** Food intake in SIRT2-WT and SIRT2-KO rats (*n*=6). **(C)** Random blood glucose of 10-week-old SIRT2-WT and SIRT2-KO rats (*n*=7). **(D)** Random plasma insulin of SIRT2-WT and SIRT2-KO rats (*n*=6). **(E)** Fasting blood glucose was measured after 16-h fasting (*n*=6). **(F)** Blood glucose excursions during a 2 g/kg i.p. glucose tolerance test (*n*=13).** (G)** Plasma insulin levels during glucose tolerance test (*n*=5).** (H)** Insulin tolerance test of SIRT2-WT and SIRT2-KO rats (*n*=11). Data are expressed as means ± SEM. ^*^P< 0.05, ^**^P< 0.01, ^***^P<0.001 *vs* WT rats.

To investigate the metabolic role of SIRT2 *in vivo*, we characterized the metabolic phenotypes of SIRT2-KO rats, in which the gene encoding SIRT2 has been genetically disrupted by CRISPR/Cas9 system (Figure S1). The body weight (Figure 1A) and food intake (Figure 1B) were comparable between SIRT2-KO and wild-type (WT) male rats. No differences were observed in random blood glucose and insulin levels between two groups (Figure 1C-D), neither was fasting blood glucose level (Figure 1E). However, 2-month-old SIRT2-KO male rats exhibited glucose intolerance (Figure 1F) and decreased insulin secretion in response to glucose challenge (Figure 1G) compared with WT rats. Knockout of SIRT2 did not alter insulin sensitivity according to insulin tolerance test (Figure 1H). SIRT2-KO female rats showed a similar phenotype (Figure S2). SIRT2 has been demonstrated to regulate hepatic gluconeogenesis via destabilizing PEPCK protein 24. However, our study revealed no significant differences in hepatic PEPCK mRNA and protein expressions at fasting and refeeding statuses between SIRT2-KO and WT male rats (Figure S3A-B), with comparable mRNA expressions of other gluconeogenic genes G6pase, Fbpase, and Foxo1 (Figure S3C). Consistent with the in-vivo result, SIRT2 inhibition by AGK2 had no impact on PEPCK protein level in primary mouse hepatocytes (Figure S3D).

### Morphology of islets and pancreatic insulin content in SIRT2 knockout rats

**Figure 2:**
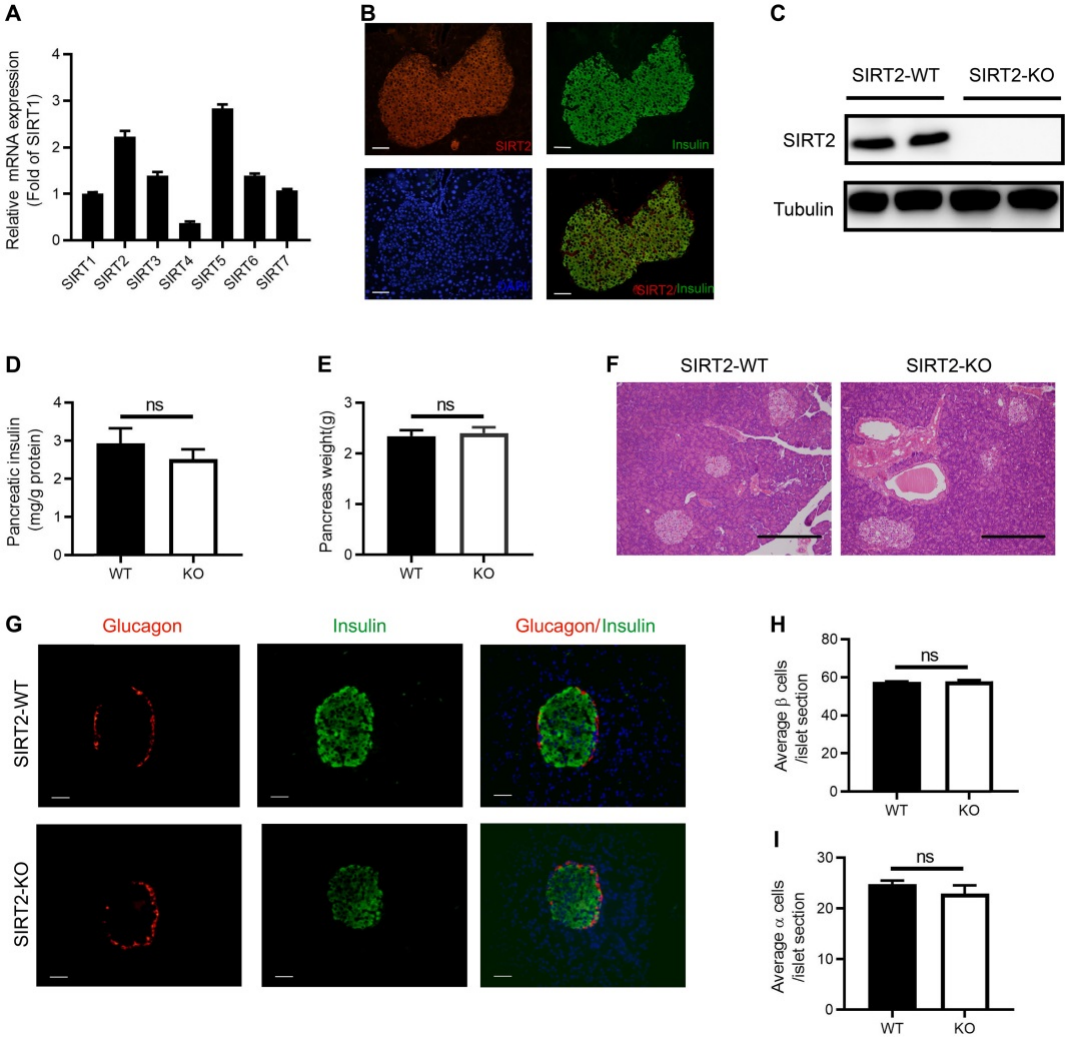
** Morphology of islet and pancreatic insulin content in SIRT2-KO rats. (A)** The mRNA expression of sirtuin family in normal islets (*n*=3).** (B)** Representative examples of normal islets stained by immunofluorescence for SIRT2 (red), insulin (green), and nuclei (DAPI, blue) (bar=40 μm).** (C)** Immunoblotting of SIRT2 in islets isolated from SIRT2-WT and SIRT2-KO rats.** (D)** Total pancreatic insulin content normalized to total protein (*n*=4-5).** (E)** Pancreatic weight of SIRT2-WT and SIRT2-KO rats (*n*=4-5).** (F)** Histological analysis of pancreas isolated from SIRT2-WT and SIRT2-KO rats. Hematoxylin-eosin staining samples were photographed at 200 magnification (bar=0.5 mm). **(G)** Representative islet of SIRT2-WT and SIRT2-KO rats stained by immunofluorescence for insulin (green), glucagon (red), and nuclei (DAPI, blue) (bar=40 μm). **(H-I)** Average number of β cells and α cells per islet section (*n*=3). Data are expressed as means ± SEM.

Similar to other sirtuin family members, the tissue distribution of SIRT2 is ubiquitous. SIRT2 has been detected in a number of tissues and organs, including liver, brain, muscle, kidney, colon, and adipose tissue 25-29. To explore the function of SIRT2 in islet, we firstly observed the abundance of SIRT2 in normal islet and found that its mRNA level was more than twice as much as SIRT1 (Figure 2A), which is the most extensively characterized family member. SIRT2 was mainly located in the cytoplasm of islet cells (Figure 2B) as in other tissue cells 22. Elevated glucose did not lead to a significant decrease in SIRT2 expression (Figure S4).

We further investigated the impact of SIRT2 knockout on pancreatic insulin content and islet morphology. Western blotting confirmed the knockout of SIRT2 gene in the islets isolated from SIRT2-KO male rats (Figure 2C). There was no significant difference in the total pancreatic insulin content (Figure 2D) or pancreas weight (Figure 2E) between SIRT2-KO and WT male rats. Islet morphology, assessed by hematoxylin and eosin (H&E) staining of pancreas sections (Figure 2F) and by immunofluorescence staining for insulin and glucagon (Figure 2G), was not appreciably altered in the SIRT2-KO rats compared with WT rats, without significant change in the average number of β-cells or α-cells per islet section (Figure 2H-I). Therefore, it is reasonable to suppose that the decreased GSIS is mainly attributed to the impaired islet secretory function.

### Knockdown or inhibition of SIRT2 decreases glucose-stimulated insulin secretion

**Figure 3:**
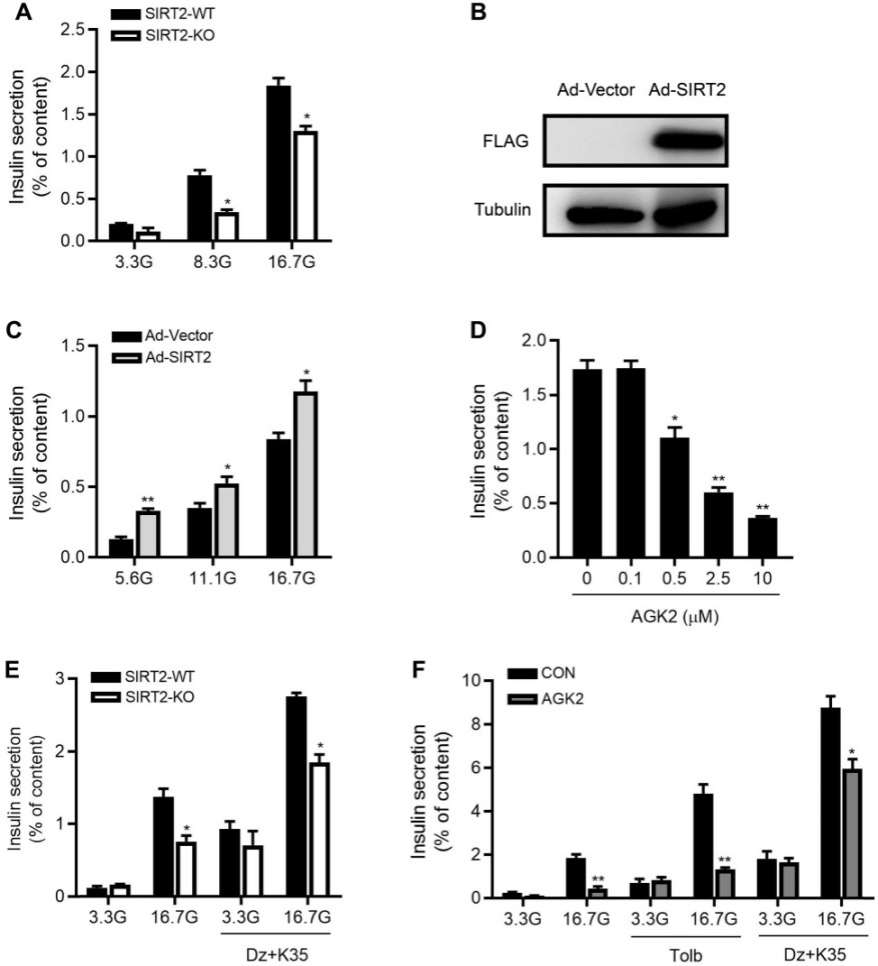
** SIRT2 promotes glucose-stimulated insulin secretion in rat islets. (A)** Islets isolated from SIRT2-WT and SIRT2-KO rats were stimulated with 3.3, 8.3 or 16.7 mM glucose for 1 h, and insulin secretion was assayed (*n*=4). **(B)** Protein level of FLAG-SIRT2 in rat islets transfected with control vector (Ad-Vector) or SIRT2-overexpressing adenovirus (Ad-SIRT2). **(C)** After transfected with Ad-Vector or Ad-SIRT2 adenovirus, islets isolated from normal rats were stimulated with 5.6, 11.1 or 16.7 mM glucose for 1 h, and insulin secretion was assayed (*n*=4). **(D)** Islets isolated from normal rats were stimulated with various concentrations of AGK2 at 16.7 mM glucose for 1 h, and insulin secretion was assayed (*n*=4). **(E)** Islets isolated from SIRT2-WT and SIRT2-KO rats were stimulated with 3.3 or 16.7 mM glucose for 1 h. The amplifying pathway of GSIS was revealed by addition of 250 μM diazoxide (Dz) and 35 mM KCl (K35) to islets (*n*=4). **(F)** Islets isolated from normal rats were incubated with or without AGK2 (3 μM) in the presence of 3.3 or 16.7 mM glucose for 1 h. The amplifying pathway of GSIS was revealed by using 500 μM tolbutamide (Tolb) or 250 μM diazoxide plus 35 mM KCl (*n*=4). Data are expressed as means ± SEM. ^*^P< 0.05, ^**^P< 0.01 *vs* control group.

To directly evaluate the effect of SIRT2 knockout on islet function, we isolated male rat islets and performed static incubation assays. Compared with control islets, insulin secretion was decreased by 56% and 29% in SIRT2-KO islets in response to 8.3 and 16.7 mM glucose (Figure 3A). After SIRT2-overexpressing adenovirus (Ad-SIRT2) was transfected into isolated WT rat islets (Figure 3B), insulin secretion was enhanced at 5.6, 11.1 and 16.7 mM glucose (Figure 3C). The pharmacological inhibition of SIRT2 by AGK2 also led to reduced GSIS in a dose-dependent manner, with a significant action at the concentration of 0.5 μM (Figure 3D). It has been widely accepted that glucose stimulates insulin secretion via generating triggering and amplifying signals in β-cells 30. We investigated whether the amplifying pathway was involved in SIRT2 deficiency-inhibited insulin secretion by treating islets with either 3.3 mM or 16.7 mM glucose in the presence or absence of KCl (35 mM) and the K_ATP_ channel activator diazoxide (250 μM). At 3.3 mM glucose, no difference was observed in insulin secretion between two groups. At 16.7 mM glucose, SIRT2-KO islets displayed a significant decrease in insulin secretion compared with control islets (Figure 3E). This was the case in AGK2-treated islets (Figure 3F), in which a high concentration of sulfonylurea tolbutamide (500 μM) was also used to explore the effect of SIRT2 on amplifying pathway. These results indicate that the amplifying pathway is involved in the regulation of SIRT2 on islet function.

### Metabolomic state of SIRT2-ablated islets

**Figure 4:**
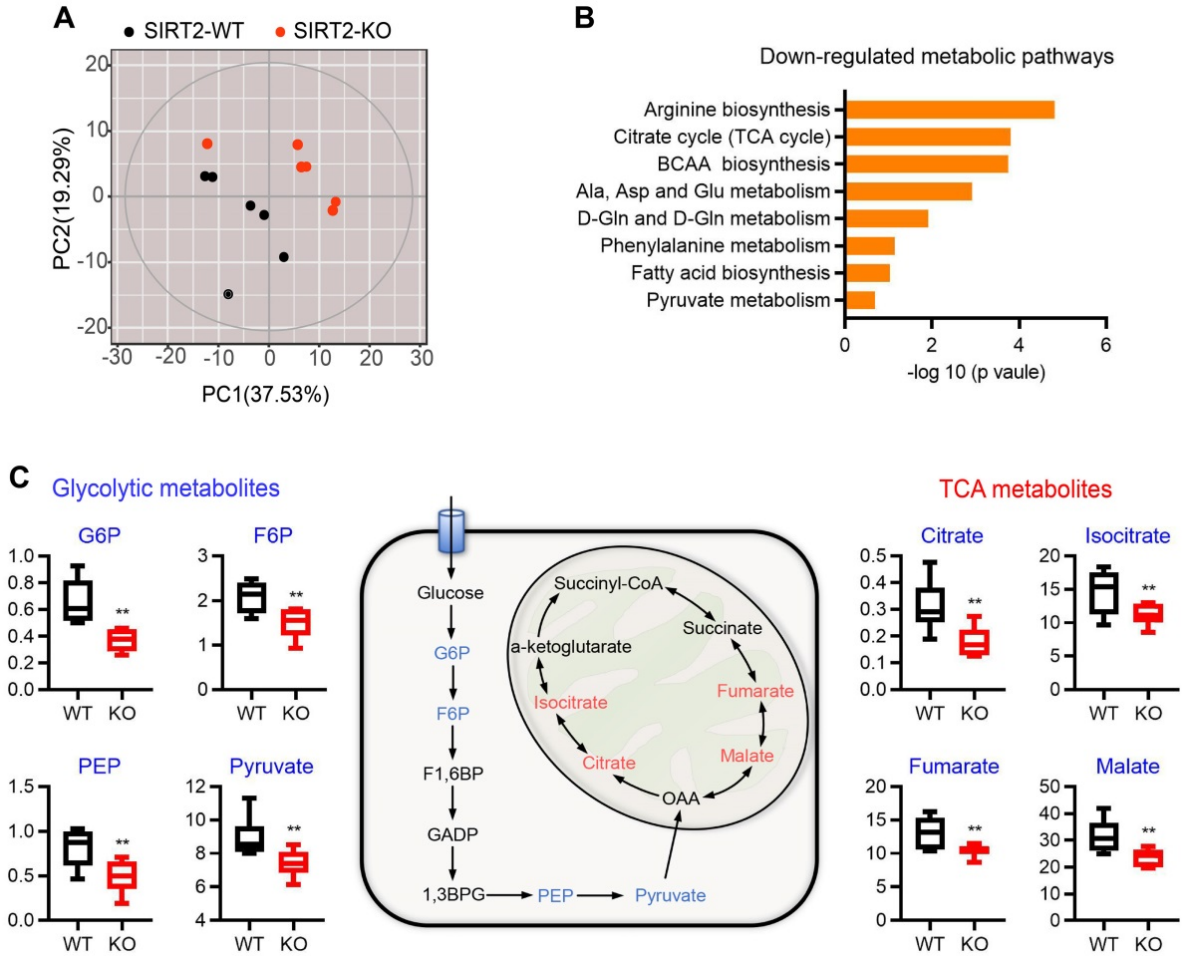
** Metabolomic change of islets isolated from SIRT2-KO rats. (A)** Overview of metabolic profiles. Metabolomics data were obtained from islet extracts (*n*=6).** (B)** Main down-regulated metabolic pathways obtained from differential metabolites of SIRT2-KO islets compared with SIRT2-WT islets.** (C)** Schematic representation of intermediates in glycolysis and TCA cycle. The relative levels of indicated metabolites are shown on both sides. Data are expressed as means ± SEM. ^*^P< 0.05, ^**^P< 0.01 *vs* WT rats.

Pancreatic islets secrete insulin in response to nutrient metabolism. In addition to triggering signal, a number of glucose-derived metabolites influence insulin secretion through the metabolic amplifying pathway 31, 32. Therefore, untargeted metabolomics analysis was performed on islets from WT and SIRT2-KO male rats to gain insight into the molecular pathways regulated by SIRT2. A total of 201 metabolites were detected and 125 of them were annotated with mammalian metabolite database JiaLib^TM^ using a strict matching algorithm incorporated in XploreMET software that uses both retention times and fragmentation patterns in the mass spectrum. The annotated metabolites were distributed into several chemical classes, including amino acid, organic acids, carbohydrates, and fatty acids. The overview of global metabolic profiles, as revealed by PCA scores plots (Figure 4A), elucidated overall profile similarities and dissimilarities between the two genotypes. Visualization of differential metabolite profiles was shown in volcano-plot (Figure S5). A total of 46 metabolites (located in the left side of volcano-plot, VIP≥ 1, p< 0.1) were decreased in SIRT2-deleted islets, while only 12 metabolites were increased (located in the right side of volcano-plot, VIP≥ 1, p< 0.1). Pathway analysis revealed that many metabolic pathways were significantly downregulated in SIRT2-KO islets, including arginine biosynthesis, TCA cycle, branched-chain amino acid (BCAA) biosynthesis, alanine/aspartate/glutamate metabolism, and pyruvate metabolism (Figure 4B). We further analyzed metabolomics results and found that glucose 6-phosphate (G6P) in SIRT2-KO islets was significantly reduced compared with control islets. G6P is derived by the phosphorylation of glucose catalyzed by the rate-limiting enzyme GCK, which is the first step of glycolysis. Besides G6P, other glycolysis intermediates such as fructose 6-phosphate (F6P), phosphoenolpyruvate, and pyruvate were also decreased by SIRT2 ablation, without increased intermediates of glycolysis. In addition, many metabolic intermediates among TCA cycle are involved in the regulation of GSIS 33. In our study, some TCA intermediates (citrate, isocitrate, fumarate, and malate) were reduced in SIRT2-KO islets while no annotated intermediates of TCA cycle were found to be significantly elevated. The change in these intermediates on a schematic of glycolysis and TCA cycle (Figure 4C) suggests that SIRT2 knockout lowers glucose-induced metabolic flux involved in GSIS.

### SIRT2 knockout or inhibition lowers glucose-stimulated oxygen consumption rate (OCR) in rat islets

**Figure 5:**
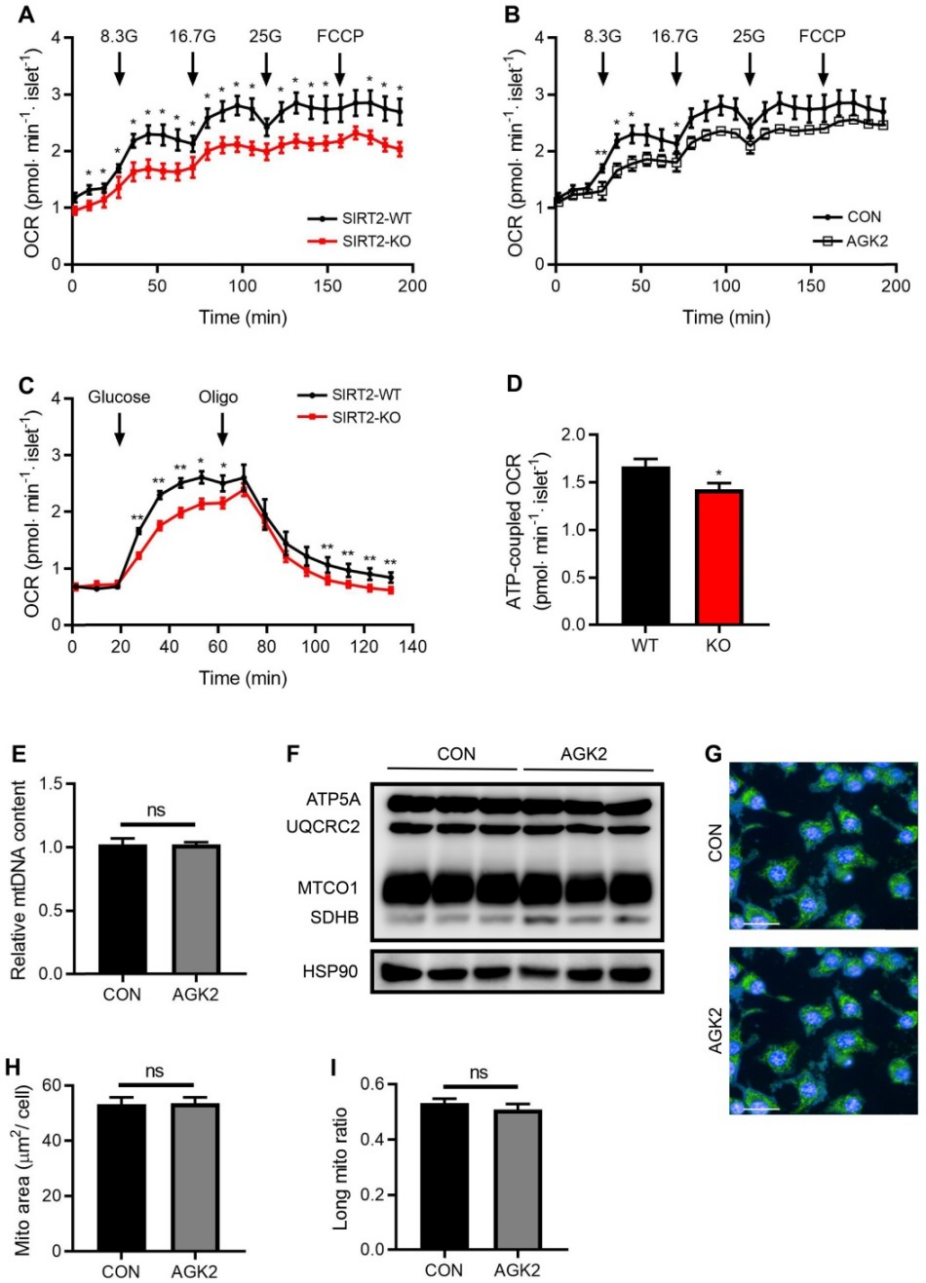
** SIRT2 knockout or inhibition decreases glucose-stimulated OCR. (A)** Oxygen consumption rate (OCR) in SIRT2-WT and SIRT2-KO islets stimulated with 8.3 mM, 16.7 mM, 25 mM glucose, and 5 µM FCCP (*n*=5). **(B)** OCR was measured in the presence of 8.3 mM, 16.7 mM, 25mM glucose or 5 µM FCCP after rat islets were pretreated with 3 μM AGK2 (*n*=5). **(C)** OCR in SIRT2-WT and SIRT2-KO islets supplemented with 16.7 mM glucose and 5 µM oligomycin (*n*=5). **(D)** ATP-coupled OCR was determined by the difference between the 16.7 mM glucose-stimulated OCR and the OCR after adding oligomycin in C (*n*=5). **(E)** mtDNA content in INS-1 cells stimulated with 3 μM AGK2 for 12 h (*n*=3). **(F)** Immunoblotting of mitochondrial genes including ATP5A, UQCRC2, MTCO1, and SDHB was detected in INS-1 cells. **(G)** INS-1 cells were stained with MitoTracker (green) as well as Hoechst (blue), and representative IFC images were shown (bar=20 μm). **(H-I)** Mitochondrial area and the ratio of long mitochondria in INS-1 cells treated with 3 μM AGK2 for 4 h. Data are expressed as means ± SEM. ^*^P< 0.05, ^**^P< 0.01 *vs* control group.

In consistent with decreased intermediates of glycolysis and TCA cycle by SIRT2 deletion, glucose supplementation-stimulated OCR was markedly decreased in SIRT2-KO islets (Figure 5A). SIRT2 inhibition by AGK2 treatment also led to a similar result in the oxygen consumption of rat islets (Figure 5B). In addition, glucose-induced mitochondrial ATP-coupled OCR was significantly reduced in SIRT2 deficient islets (Figure 5C-D). A critical role of the mitochondria is to generate ATP from ADP and organophosphate via oxidative phosphorylation. Previous study demonstrates that SIRT2 functions as a regulator of autophagy/mitophagy to maintain mitochondrial biology in mouse neurons and embryonic fibroblasts 20. We further investigated whether SIRT2 exerted a similar action in β-cell mitochondria. mtDNA content and protein levels of TCA-related enzymes (ATP5A, UQCRC2, MTCO1, and SDHB) showed no changes in INS-1 cells after AGK2 treatment (Figure 5E-F). In addition, the mitochondrial morphology in INS-1 cells was observed using MitoTracker staining. AGK2 treatment had no significant impact on mitochondrial morphology as shown in Figure 5G. Further analysis demonstrated that the mean mitochondrial area and ratio of long mitochondria in INS-1 cells were comparable between the two groups (Figure 5H-I). Therefore, it is likely that SIRT2-regulated OCR on glucose stimulation is independent of mitochondrial function.

### SIRT2 inhibition increases GKRP protein level and its interaction with GCK

**Figure 6:**
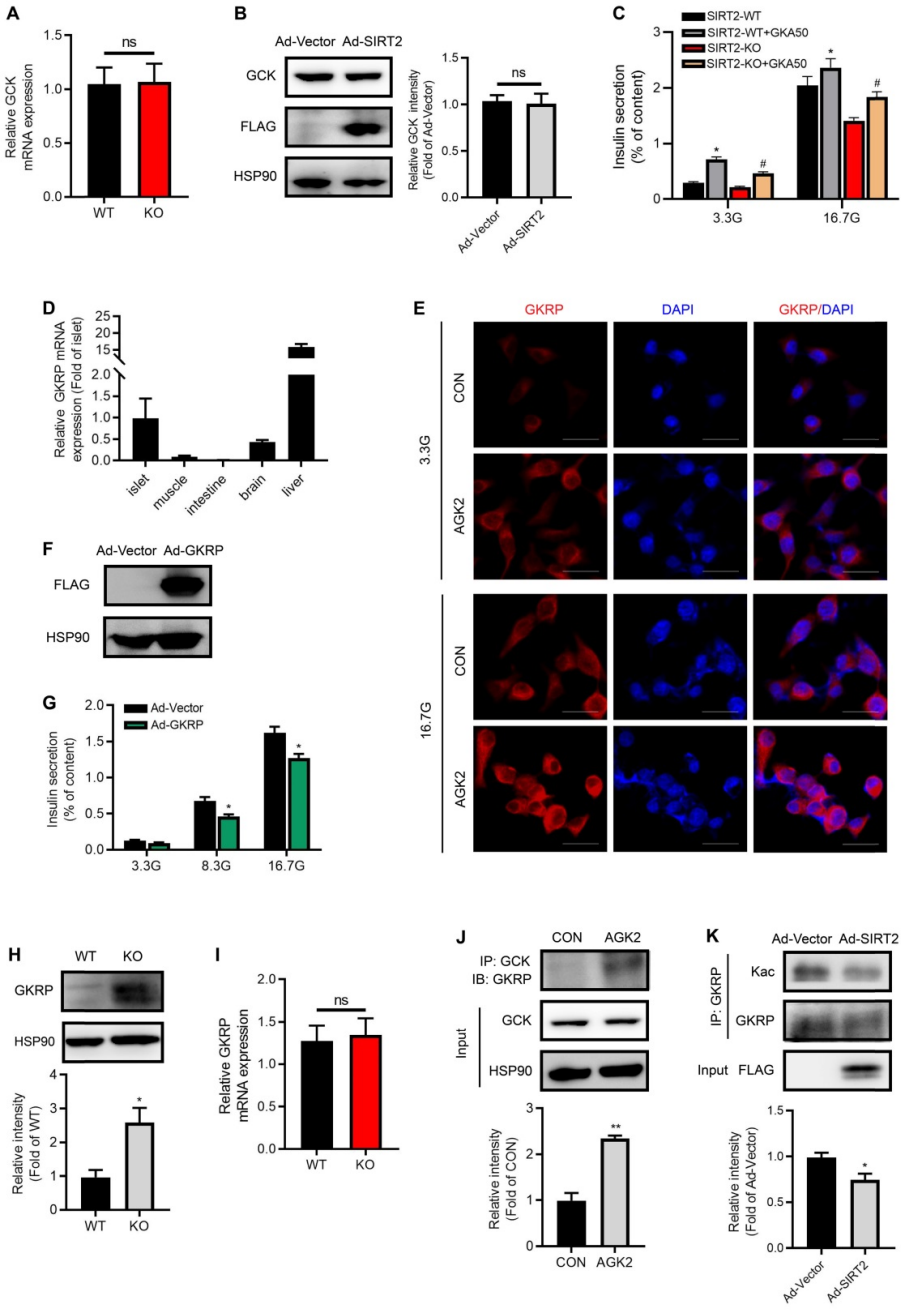
** SIRT2 inhibition stabilizes GKRP protein and enhances its binding to GCK. (A)** mRNA levels of GCK in the islets isolated from SIRT2-WT and SIRT2-KO rats (*n*=3). **(B)** Protein expression of GCK in INS-1 cells transfected with Ad-SIRT2 or Ad-Vector adenovirus for 36 h. **(C)** Islets isolated from SIRT2-WT and SIRT2-KO rats were incubated with 3 µM GKA50 in the presence of 3.3 or 16.7 mM glucose for 1 h, and insulin secretion was assayed (*n*=4).** (D)** mRNA levels of GKRP in different tissues (*n*=6). **(E)** INS-1 cells treated with 3 μM AGK2 for 6 h in the presence of 3.3 or 16.7 mM glucose were stained by immunofluorescence for GKRP (red) and nuclei (DAPI, blue) (bar=20 μm). **(F)** Protein level of GKRP in rat islets transfected with Ad-Vector or GKRP-overexpressing adenovirus (Ad-GKRP). **(G)** Rat islets transfected with Ad-Vector or Ad-GKRP adenovirus were stimulated with 3.3, 8.3 or 16.7 mM glucose for 1 h, and insulin secretion was assayed (*n*=4). **(H-I)** Protein and mRNA expressions of GKRP in the islets isolated from SIRT2-WT and SIRT2-KO rats (*n*=3). **(J)** The interaction between GCK and GKRP was detected in INS-1 cells treated with 3 μM AGK2 for 6 h. **(K)** After INS-1 cells were transfected with Ad-Vector or Ad-SIRT2 adenovirus, cell lysates were immunoprecipitated with GKRP antibody and subjected to Western blot with anti-acetyllysine antibody. Data are expressed as means ± SEM. ^*^P< 0.05, ^**^P< 0.01 *vs* control group. ^#^*P*< 0.05 *vs* SIRT2-KO group.

We further sought to explore SIRT2 effectors in the process of glycolysis. In spite of low G6P level observed in SIRT2-KO islets, no significant changes for GCK mRNA and protein expressions were observed after SIRT2 ablation or overexpression (Figure 6A-B). These results drew our attention to the activity of GCK. As shown in Figure 6C, the glucokinase activator GKA50 antagonized SIRT2 deficiency-impaired GSIS, suggesting that SIRT2 may regulate GCK activity so as to control the first step in glycolysis after glucose enters β-cells. GKRP has been demonstrated to inhibit the GCK activity competitively with the substrate glucose 34, 35. Previous study reported that GKRP was expressed only in liver 36. Our study showed that GKRP mRNA level in islet was much less than that in liver, but more than twice as much as that in brain (Figure 6D), where the expression of GKRP was previously proved 37. GKRP expression was hardly detectable in muscle and intestine (Figure 6D). In INS-1 β-cell line, GKRP protein was mainly located in the cytoplasm at both low and high glucose concentrations as shown by immunofluorescence staining (Figure 6E), inconsistent with its distribution in hepatocytes, where GKRP binds GCK and sequesters the enzyme to the nucleus at low glucose. Elevated glucose concentration leads to the disruption of GKRP-GCK complex, enabling the migration of GCK and GKRP to the cytoplasm 38. AGK2 increased the abundance of GKRP protein in INS-1 cells at both 3.3 and 16.7 mM glucose (Figure 6E). Insulin secretory response to glucose stimulation was significantly reduced by GKRP overexpression (Figure 6F-G). Similarly, GKRP protein expression in SIRT2-KO islets was much higher than that in control islets (Figure 6H). However, the change in GKRP mRNA expression level was not observed (Figure 6I), indicating a possibility that SIRT2 regulates GKRP protein expression at posttranslational level. Similar to the result in hepatocytes previously described 39, the interaction of GCK with GKRP was enhanced by the treatment of AGK2 (Figure 6J). GKRP has been reported to be a direct target of SIRT2 in mice liver 39. We wondered whether its acetylation was also regulated by SIRT2 in islet β-cells. Adenovirus-mediated SIRT2 overexpression significantly decreased GKRP acetylation level in INS-1 cells (Figure 6K). Collectively, SIRT2 knockout or inhibition markedly enhances GKPR protein expression and its interaction with GCK in islets, leading to a decrease in GCK activity.

### SIRT2 inhibition promotes GKRP protein stability and ALDO protein degradation

**Figure 7:**
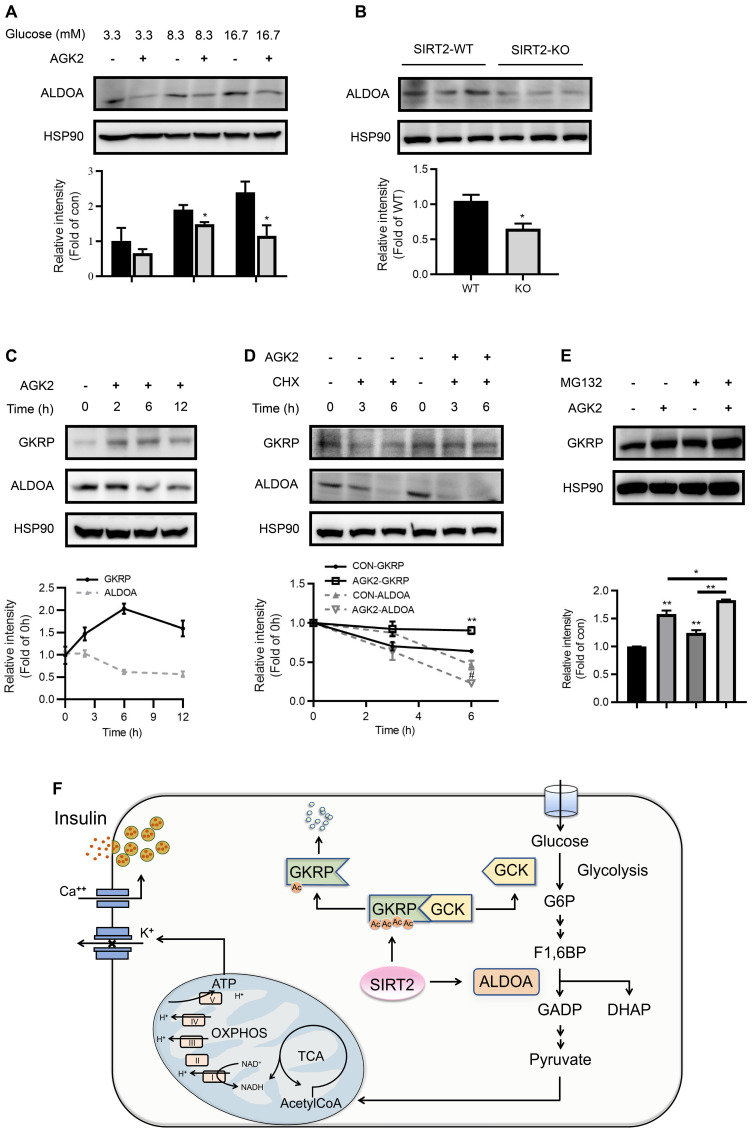
** SIRT2 inhibition promotes GKRP protein stability and ALDOA protein degradation. (A)** ALDOA protein level in INS-1 cells treated with 3 μM AGK2 in the presence of 3.3, 8.3 or 16.7 mM glucose. **(B)** Protein expression of ALDOA in the islets isolated from SIRT2-WT and SIRT2-KO rats. **(C)** GKRP and ALDOA protein expressions in INS-1 cells incubated with 3 μM AGK2 for the indicated time points. **(D)** GKRP and ALDOA protein expressions in INS-1 cells exposed to 3 μM AGK2 and 10 μg/ml cycloheximide (CHX) for 3 and 6 h. **(E)** GKRP protein expression in INS-1 cells treated with or without 3 μM AGK2 and 10 μM MG132 for 6 h. Signal intensity was quantified by image J software for statistical comparison. **(F)** The schematic illustration summarizes the role of SIRT2 in glucose-stimulated insulin secretion. GCK acts as a glucose sensor in the β-cells by converting glucose to G6P, which eventually yields ATP through glycolysis, TCA cycle, and oxidative phosphorylation for triggering insulin secretion. GKRP binds and inactivates GCK. SIRT2 inhibits ALDOA protein degradation and decreases GKRP protein stability by decreasing its acetylation level, thereby disrupting the GKRP-GCK complex and promoting glycolytic flux and insulin secretion. Data are expressed as means ± SEM. ^*^P< 0.05, ^**^P< 0.01 *vs* control group. ^#^*P*< 0.05 *vs* CON-ALDOA group.

Aldolase is a crucial enzyme in glycolysis responsible for the reversible aldol reaction of fructose-1,6-bisphosphate (FBP) to glyceraldehyde-3-phosphate and dihydroxyacetone phosphate. Aldolase A (ALDOA) revealed a significantly higher expression in rat islets compared with other two aldolase isozymes (B and C) (Figure S6). In contrast to GKRP, the protein expression of ALDOA was suppressed by AGK2 (Figure 7A). Islets isolated from SIRT2-KO rats also exhibited a significant decrease in ALDOA protein level compared with control islets (Figure 7B). The inverse changes in GKRP and ALDOA mediated by AGK2 proved to be a rapid progress, which occurred in INS-1 cells treated with AGK2 for just 6 h. The increase of GKRP protein level could even be observed just after 2-h treatment of AGK2 (Figure 7C). Moreover, SIRT2 pharmacological inhibition by AGK2 significantly prevented the degradation of GKRP protein in INS-1 cells in the presence of protein synthesis inhibitor cycloheximide (CHX). On the other hand, AGK2 dramatically shortened the half-life of ALDOA in INS-1 cells (Figure 7D). Combined treatment of AGK2 and the proteasome inhibitor MG132 exerted a synergistic effect on the prevention of GKRP protein degradation in INS-1 cells (Figure 7E). Taken together, these results indicate that knockout or inhibition of SIRT2 lessens glycolytic flux via promoting GKRP protein stability and ALDOA protein degradation.

## Discussion

Abnormalities in islet function are critical in defining the risk and development of type 2 diabetes 40. SIRT2 is a key regulator of cytoplasmic protein acetylation status 41, but its biological function has not been examined in pancreatic β-cells. The present study demonstrated that genetic deletion or pharmacological inhibition of SIRT2 led to impaired insulin secretion from rat islets, in which the metabolic amplifying pathway was involved. SIRT2 ablation significantly reduced glucose-induced glycolytic influx in β-cells via stabilizing GKRP protein and promoting its binding to GCK. Therefore, glucose intolerance in SIRT2-KO rats is mainly attributed to impaired GSIS due to the deacceleration of glucose metabolism in islets.

As a fuel-sensing molecule, SIRT2 expression is induced during low-energy status and repressed under energy excess condition 42. In contrast, SIRT2 expression displayed no change in islets exposed to high glucose in our study. SIRT2 protein expressions in visceral WAT from human obese subjects and high-fat diet-fed mice were decreased compared with lean controls 43. It has been demonstrated that SIRT2 inhibits adipocyte differentiation through deacetylating Foxo1 and inhibiting PPARγ 44. SIRT2 deacetylates and destabilizes ATP-citrate lyase (ACLY), a key enzyme for *de novo* fatty acid synthesis 45. Overexpression of SIRT2 promotes fatty acid oxidation via deacetylating PGC-1α 43. These results suggest that enhancing SIRT2 activity is beneficial for obesity-related disease, such as type 2 diabetes mellitus, nonalcoholic fatty liver, and metabolic syndrome. However, our study exhibited a comparable body weight between SIRT2-KO and WT rats.

Genetic polymorphism of SIRT2 has been reported to associate with diabetes development 46. However, previous studies displayed contradictory results about the role of SIRT2 in maintaining glucose homeostasis. Watanabe et al. 39 reported that hepatic SIRT2 knockdown by tail vein injection of adenovirus expressing SIRT2 siRNA resulted in impaired glucose tolerance by suppressing hepatic glucose uptake. Another study showed a contrary result, in which SIRT2 silence decreased blood glucose level in mice by promoting PEPCK acetylation and degradation 24. However, our study showed that neither genetic knockout nor pharmacological inhibition of SIRT2 altered the expression levels of hepatic gluconeogenic genes. It was reported that glucose intolerance was markedly improved in SIRT2-KO mice 47. But in another study, SIRT2-KO did not alter glucose disposal in mice fed with chow diet 48. In this current study, the deletion of SIRT2 led to glucose intolerance in chow diet-fed rats. In addition, there also exist conflicting results regarding the function of SIRT2 on insulin sensitivity. It has been demonstrated that SIRT2 overexpression improves insulin sensitivity in insulin-resistant hepatocytes 49 and enhances insulin-activated Akt signaling in HeLa cells and 3T3-L1 preadipocytes 50. Lantier et al. 48 showed that SIRT2 deletion alone was sufficient to impair muscle insulin sensitivity in chow diet-fed mice and high fat diet-induced liver insulin resistance was worsened by SIRT2 KO. But in C2C12 muscle cells, SIRT2 negatively regulates insulin sensitivity and glucose uptake 51. In SIRT2-KO mice reported by Belman et al. 47, insulin-stimulated whole body glucose uptake was greatly enhanced. In our study, the insulin sensitivity was comparable between SIRT2-KO and WT rats, but blood insulin level after glucose loading was markedly decreased in SIRT2-KO rats, which may explain glucose intolerance.

Among the Sirtuin family, SIRT1 and SIRT6 are the positive regulators of insulin secretion 12, 13, 18 while two mitochondrial deacetylases SIRT3 and SIRT4 negatively regulate insulin secretion 8, 16. However, the role of SIRT2 in islet function still remains enigmatic. In the present study, the islets isolated from SIRT2-KO rats secreted less insulin in response to glucose challenge and the overexpression of SIRT2 in rat islets enhanced GSIS, indicating that SIRT2 is a direct regulator of islet function. The triggering pathway is essential for glucose-induced insulin secretion, which has been well characterized 30. Glucose-stimulated amplitude of insulin secretion largely depends on the amplifying pathway that does not raise intracellular Ca^2+^ concentration further, but metabolic molecules augment the action of triggering Ca^2+^ on exocytosis 52. Our study showed that genetic knockout or pharmacological inhibition of SIRT2 disrupted the metabolic amplifying pathway of GSIS, suggesting the involvement of metabolites in SIRT2-modulated insulin secretion.

The pancreatic β-cells sense circulating levels of nutrients to secrete insulin. Glucose is the primary stimulus for insulin secretion, whose metabolism in β-cells is achieved by tightly linking glycolysis with mitochondrial metabolism 53. In this current study, decreased glucose-stimulated OCR and universal reduction of glycolytic and TCA intermediates with unaffected mitochondria were detected in SIRT2-KO islets, implicating the influx of insufficient substrate. Unlike most other tissues where metabolism primarily responds to nerve or hormonal stimulation and energy expenditures, glucose metabolism in β-cells is governed by substrate availability 54. This unique feature is possible because: (1) glucose transport into β-cells is not limiting 55. (2) glycolysis is controlled by GCK, lacking any form of feedback control 56. (3) The use of G6P by other pathways (e.g., pentose phosphate pathway and glycogen synthesis) is very limited in islets 57. Thereby the cellular levels of glucose as well as the expression and activity of GCK primarily determine glucose usage and regulate insulin secretion in islets. SIRT2 ablation lowered the concentrations of G6P and subsequent intermediates in islet without changing GCK expression, indicating a possibility that SIRT2 plays a regulatory role in GCK activity. The GCK activator did rescue SIRT2 knockout-impaired GSIS, suggesting that SIRT2 facilitates GSIS by promoting the activity of GCK.

GKRP functions as an allosteric inhibitor of glucokinase and as a metabolic sensor 58. GCK is bound to GKRP at low glucose status, resulting in its inactivation. Glucose induces GCK-GKRP dissociation and GCK activation 34. GKRP was reported to be only expressed in liver, but one study described the existence of a brain GKRP and its functional interaction with GCK 37. In our study, GKRP protein expression was detectable in islet β-cells, which was significantly induced by SIRT2 inhibition. Park J et al. 59 and Watanabe H et al. 39 implicate that SIRT2-dependent GKRP deacetylation facilitates the dissociation of GKRP from GCK, thereby promoting GCK activity in the liver. Our study exhibited a similar result that SIRT2 overexpression deacetylated GKRP in INS-1 cells and SIRT2 inhibition by AGK2 enhanced the interaction of GKRP and GCK. However, unlike the result in liver that SIRT2 failed to affect GKRP expression 39, SIRT2 directly regulated the protein stability of GKRP in islets. Thus, it is possible that SIRT2 inhibition-mediated GKRP protein stability is linked to its acetylation.

A recent study showed that knockdown of SIRT2 in fibroblasts considerably increased the acetylation levels of four glycolytic enzymes (ALDOA, GAPDH, PGK1, and ENO1) and enhanced their enzymatic activities, without affecting the total amount of enzymes 60. In contrast to the result, we found that SIRT2 knockout or inhibition decreased the protein level of ALDOA via destabilizing its protein, which acted in concert with GKRP to reduce the glycolytic flux for insulin secretion. The effect of SIRT2 on glycolysis in islets and liver seems contrast to that in fibroblasts. After all, both islets and liver are the most metabolically relevant tissues, in which nutrients metabolism is quite different from that in other tissues concerning their vital roles in maintaining energy homeostasis. The opposing role of SIRT2 in different tissues needs further investigation.

In summary, this study demonstrates a positive role of SIRT2 played in islet function. Increased protein stability of GKRP followed by reduced glycolytic flux is responsible for the impaired GSIS in SIRT2-KO islets (Figure 7F). Since GKRP disruptors have been expected as a potential new class of glucose-lowering drugs 61, the action of GKRP in islets should be taken into account for these agents in treating type 2 diabetes mellitus.

## Supplementary Material

Supplementary figures.

## Abbreviations

SIRT2: NAD-dependent deacetylase sirtuin 2
GSIS: glucose-stimulated insulin secretion
GKRP: glucokinase regulatory protein
GCK: glucokinase
ALDOA: aldolase A
TCA: tricarboxylic acid
KATs: lysine acetyltransferases
KDACs: lysine deacetylases
H&E: hematoxylin and eosin
G6P: glucose 6-phosphate
F6P: fructose 6-phosphate
FBP: fructose-1,6-bisphosphate
CHX: cycloheximide
FOXO1: forkhead box protein O1
PPARγ: peroxisome proliferator-activated receptor γ
ACLY: ATP-citrate lyase
PGC-1α: peroxisome proliferator-activated receptor gamma coactivator 1-α
PEPCK: phosphoenolpyruvate carboxykinase
G6pase: glucose 6-phosphatase
Fbpase: fructose 1,6-bisphosphatase
