# Supplementary material for: SIRT2 ablation inhibits glucose-stimulated insulin secretion through decreasing glycolytic flux
Source: Theranostics. 2021 Mar 4;11(10):4825–38. doi: 10.7150/thno.55330 (PMC7978320; doi:10.7150/thno.55330)
Supplement: Supplementary file 1 — Supplementary figures. [file thnov11p4825s1.pdf]

Figure S1. Genotyping products of SIRT2-WT and SIRT2-KO rats. Product sizes were 540 and 380 bp for WT and KO alleles, respectively.

Figure S2. Metabolic phenotype in female SIRT2-WT and SIRT2-KO rats. (A) Body weight curves of SIRT2-WT and SIRT2-KO rats (*n*=6). **(B)** Food intake in SIRT2-WT and SIRT2-KO rats at ~15 weeks of age (*n*=4). **(C)** Random blood glucose (*n*=15). **(D)** Blood glucose levels in body weight-matched rats were measured at the indicated times after 16-h fasting during IPGTT(*n*=14). Data are expressed as means ± SEM. $^{**}P< 0.01$, $^{***}P< 0.001$ *vs* WT rats.

Figure S3. SIRT2 knockout or inhibition has no impact on gluconeogenic gene expressions. (A-B) mRNA and protein expressions of PEPCK in the liver of SIRT2-WT and SIRT2-KO male rats under the status of fasting or refeeding (*n*=4). **(C)** mRNA expression of gluconeogenic genes in the liver of SIRT2-WT and SIRT2-KO male rats (*n*=4). **(D)** Protein level of PEPCK in primary mouse hepatocytes treated with 3 μM AGK2. Data are expressed as means ± SEM. $^{***}P< 0.001$ *vs* WT rats.

Figure S4. mRNA level of SIRT2 in rat islets at low and high glucose concentrations.

Figure S5. Visualization of metabolite profiles is presented by volcano-plots. Up-regulated and down-regulated metabolites in SIRT2-KO islets versus SIRT2-WT islets were distributed in the right and left side, respectively (VIP> 1, blue points indicate P<0.1, green points indicate P<0.05).

Figure S6. mRNA expressions of three aldolase isozymes (A, B, and C) in rat islets. Data are expressed as means ± SEM. $^{***}P< 0.001$ *vs* ALDOA.

Figure S1

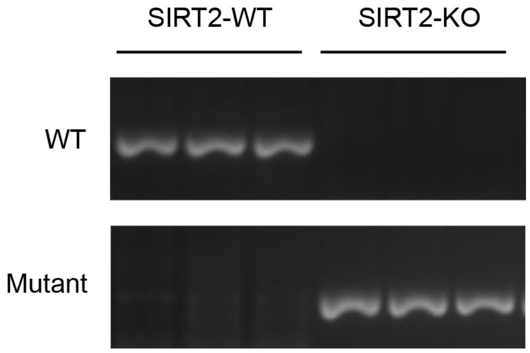

Figure S2

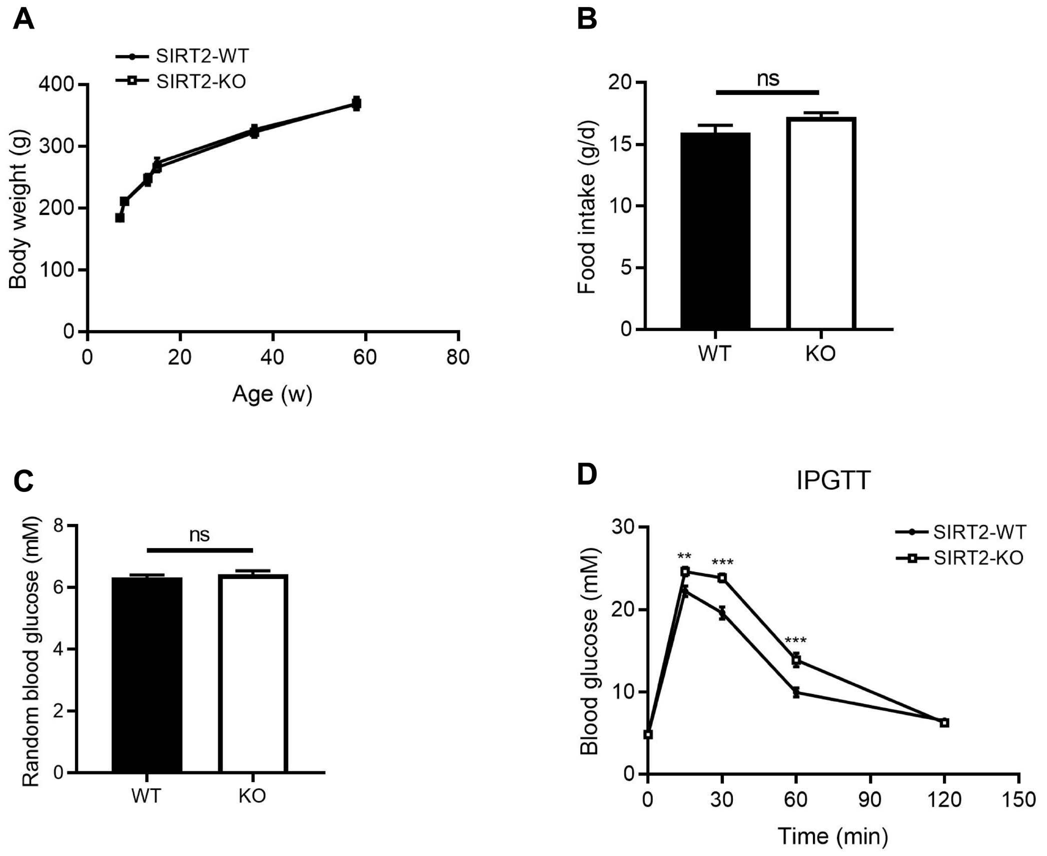

Figure S3

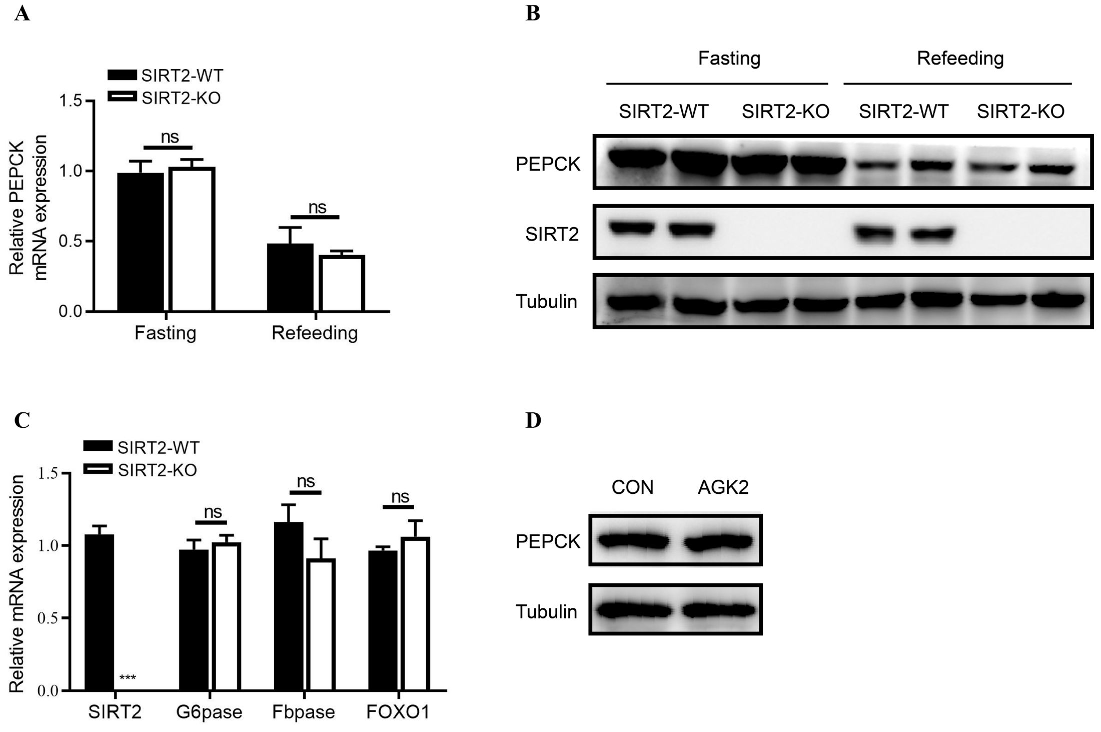

Figure S4

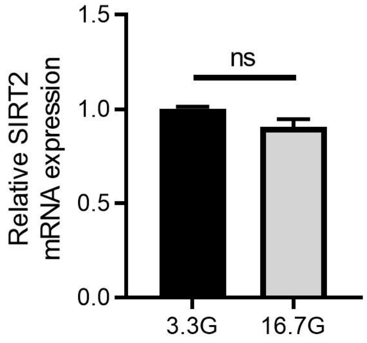

Figure S5

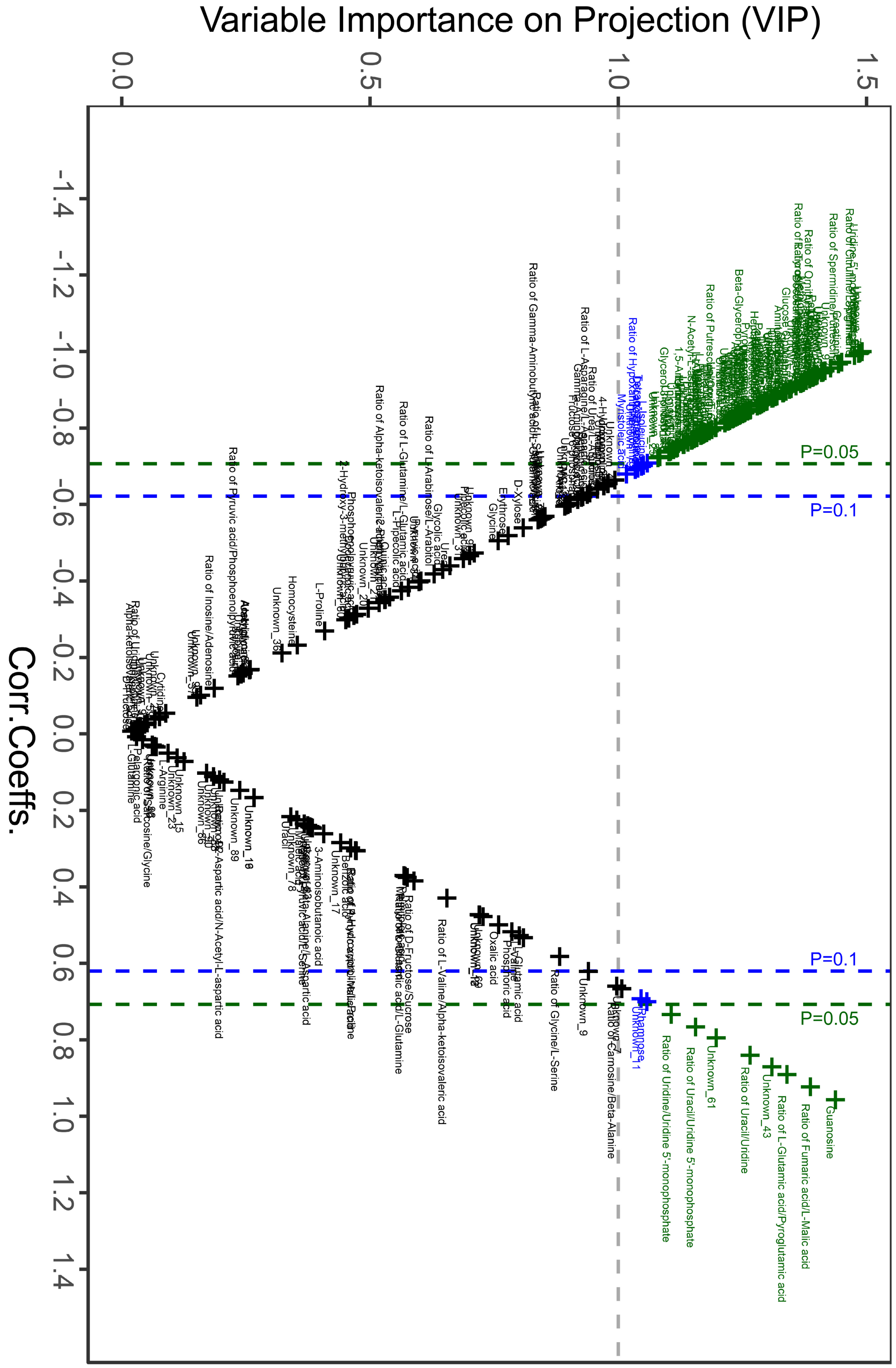

Variable Importance on Projection (VIP)
0.0
0.5
1.0
1.5
Corr.Coeffs.
-1.4
-1.2
-1.0
-0.8
-0.6
-0.4
-0.2
0.0
0.2
0.4
0.6
0.8
1.0
1.2
1.4
P=0.05
P=0.1
P=0.1
P=0.05
Guanosine
Ratio of Fumaric acid/L-Malic acid
Ratio of L-Glutamic acid/Pyroglutamic acid
Unknown_43
Ratio of Uracil/Uridine
Unknown_61
Ratio of Uracil/Uridine 5'-monophosphate
Ratio of Uridine/Uridine 5'-monophosphate
Ratio of Carnosine/Beta-Alanine
Unknown_9
Ratio of Glycine/L-Serine
Phosphoric acid
Oxalic acid
Ratio of L-Valine/Alpha-ketoisovaleric acid
Ratio of D-Fructose/Sucrose
3-Aminoisobutanoic acid
Unknown_17
Unknown_78
Uracil
Unknown_89
Unknown_18
Ratio of L-Aspartic acid/N-Acetyl-L-aspartic acid
L-Arginine
Unknown_23
Unknown_15
Pelargonic acid
L-Glutamine
Cytidine
Ratio of Inosine/Adenosine
Ratio of Pyruvic acid/Phosphoenolpyruvic acid
Unknown_36
Homocysteine
L-Proline
Ratio of L-Glutamine/L-Glutamic acid
L-Pipecolic acid
Ratio of L-Arabinose/L-Arabitol
Glycolic acid
Urea
Glycine
Erythrose
D-Xylose
Myristoleic acid

Figure S6

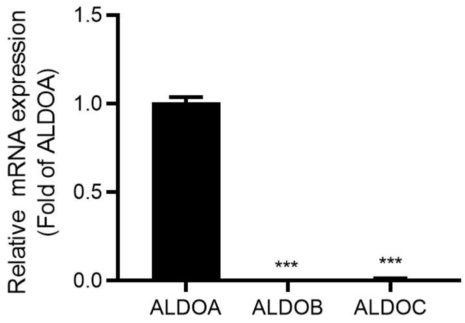
Relative mRNA expression
(Fold of ALDOA)
1.5
1.0
0.5
0.0
***
***
ALDOA
ALDOB
ALDOC
